# Recovery of Corneal Endothelial Cells from Periphery after Injury

**DOI:** 10.1371/journal.pone.0138076

**Published:** 2015-09-17

**Authors:** Sang Ouk Choi, Hyun Sun Jeon, Joon Young Hyon, Yun-Jung Oh, Won Ryang Wee, Tae-young Chung, Young Joo Shin, Jeong Won Kim

**Affiliations:** 1 Department of Ophthalmology, Hallym University College of Medicine, Seoul, Republic of Korea; 2 Department of Ophthalmology, Seoul National University Bundang Hospital, Seongnam, Gyeonggi, Korea; 3 Department of Ophthalmology, Seoul National University College of Medicine, Seoul, Republic of Korea; 4 Department of Ophthalmology, Samsung Medical Center, Sungkyunkwan University School of Medicine, Seoul, Republic of Korea; 5 Department of Pathology, Hallym University College of Medicine, Seoul, Republic of Korea; Cedars-Sinai Medical Center; UCLA School of Medicine, UNITED STATES

## Abstract

**Background:**

Wound healing of the endothelium occurs through cell enlargement and migration. However, the peripheral corneal endothelium may act as a cell resource for the recovery of corneal endothelium in endothelial injury.

**Aim:**

To investigate the recovery process of corneal endothelial cells (CECs) from corneal endothelial injury.

**Methods:**

Three patients with unilateral chemical eye injuries, and 15 rabbit eyes with corneal endothelial chemical injuries were studied. Slit lamp examination, specular microscopy, and ultrasound pachymetry were performed immediately after chemical injury and 1, 3, 6, and 9 months later. The anterior chambers of eyes from New Zealand white rabbits were injected with 0.1 mL of 0.05 N NaOH for 10 min (NaOH group). Corneal edema was evaluated at day 1, 7, and 14. Vital staining was performed using alizarin red and trypan blue.

**Results:**

Specular microscopy did not reveal any corneal endothelial cells immediately after injury. Corneal edema subsided from the periphery to the center, CEC density increased, and central corneal thickness decreased over time. In the animal study, corneal edema was greater in the NaOH group compared to the control at both day 1 and day 7. At day 1, no CECs were detected at the center and periphery of the corneas in the NaOH group. Two weeks after injury, small, hexagonal CECs were detected in peripheral cornea, while CECs in mid-periphery were large and non-hexagonal.

**Conclusions:**

CECs migrated from the periphery to the center of the cornea after endothelial injury. The peripheral corneal endothelium may act as a cell resource for the recovery of corneal endothelium.

## Introduction

The corneal endothelium lines the posterior surface of the cornea and faces the anterior chamber [1]. It provides a surface for metabolic exchange between the cornea and aqueous humor [2,3] and forms a leaky barrier, allowing aqueous humor to pass through the cell junctions [3]. Corneal endothelial cells (CECs) are monolayers of closely interdigitated hexagonal cells arranged in a mosaic pattern [1,2], which play an essential role in maintaining corneal deturgescence and transparency [1]. They also control the active transport of fluid out of the cornea via the activity of the Na^+^/K^+^-ATPase and the bicarbonate-dependent Mg^2+^-ATPase ion pump resulting in stromal deturgescence [1,3,4]. Both the barrier and the “pump” functions of the endothelium are essential for maintaining the relatively dehydrated state of the stroma required for transparency [1,4,5].

The wound healing processes of CECs have been reported to be different from those of other cell types [1,6]. In contrast to the corneal epithelium, wound healing of the endothelium occurs mainly through cell enlargement and migration [1,5,7]. Diseased or damaged endothelial cells are replaced with adjacent endothelial cells [1,7] and the enlargement of endothelial cells involves polyploidization [8]. In rat corneal transplantation, it has been reported that host-derived endothelial cells migrate to grafts, suggesting that the peripheral corneal endothelium has a high regenerative capacity [9,10].

Extensive injury of corneal endothelium leads to permanent corneal swelling and bullous keratopathy [11]. Chemical injury of the ocular surface can frequently result in severe inflammation, epithelial necrosis, and limbal stem cell deficiency [12]. Deep penetration of chemicals into anterior segments can cause corneal endothelial damage and corneal edema [12,13]. However, corneal endothelial regeneration in the extensive corneal endothelial damages after chemical injury has not yet been reported. This study investigated the regeneration of corneal endothelial cells in the healing process of corneal endothelial injury.

## Patients and Methods

### Patients with chemical injury

An observational case series was conducted at the Department of Ophthalmology, Hallym University Kangnam Sacred Heart Hospital, Seoul, Korea. All procedures conformed to the Declaration of Helsinki. The study protocol was approved by the Institutional Review Board of Hallym University Medical Center. Three patients who sustained chemical eye injuries were included in this study. Three patients in this manuscript have given written informed consent to publish these case details. Slit lamp examination, specular microscopy, and ultrasound pachymetry (USP) were performed in patients with unilateral chemical eye injury immediately after chemical injury and 1, 3, 6, and 9 months later.

### Slit lamp biomicroscopy

Slit lamp microscopy was used to evaluate corneal changes including corneal edema, neovascularization, and Descemet’s membrane folds.

### Corneal endothelial cell measurements using non-contact specular microscopy

A non-contact specular microscope Topcon SP-3000P (Topcon Corp., Tokyo, Japan) was used to perform corneal endothelial cell measurements. The patient’s head was positioned against the headband and chin rest, and the patient was instructed to look straight ahead to the fixation targets. Images of the central cornea area were captured using the automatic-mode low flash intensity. Each image was taken after correct positioning of the alignment dot, circle, and bar on the screen. Endothelial cell morphology analyses were performed using automated measurements with the retracing method in the IMAGEnet® software program (Topcon), the manufacturer’s built-in image analysis software. The CECD was estimated using the mean of three consecutive measurements and expressed as number of cells per mm^2^. The images were then printed with the analyzed data.

### Central corneal thickness measurement using ultrasound pachymetry

For USP (Tomey SP-100, Tomey Ltd, Tokyo, Nagoya, Japan) measurements, the probe was first disinfected with an alcohol swab and the seated patient was instructed to fixate on a distant target. One drop of a topical anesthetic (proparcaine hydrochloride 0.5%) was instilled in the conjunctival fornix of the test eye. The ultrasound probe was then aligned perpendicular to the center of the cornea and gently placed in contact with the cornea. Three consecutive readings were taken for each patient and the calculated CCT was expressed in μm.

### Animals

All procedures were performed according to the Association for Research in Vision and Ophthalmology (ARVO) Statement for the Use of Animals in Ophthalmic and Vision Research. This study was approved by the Institutional Animal Care and Use Committee (IACUC) of Seoul National University Bundang Hospital. Female New Zealand white rabbits, with an approximate body weight of 1.8 kg, were purchased from Nara-Biotech Animal Company (Seoul, Republic of Korea). These rabbits were maintained under constant conditions of 23°C and 60% relative humidity, and exposed to a 12 h day-night cycle (8 A.M.–8 P.M.; 8 P.M.–8 A.M.).

### Corneal endothelial injury and central corneal thickness measurements

Fifteen rabbits were divided into two groups: those exposed to NaOH (n = 12) and controls (n = 3). Anesthesia was administrated by intramuscular injections of tiletamine and zolazepam (30 mg/kg; Zoletil 50; Virbac; Carros, France) and xylazine hydrochloride (5 mg/kg); proparacaine eyedrops (Alcaine; Alcon; Fort Worth, TX, USA) were used as topical anesthesia. The anterior chamber of the right eye of each rabbit was treated with 0.1 mL of 0.05 N NaOH for 10 min followed by administration of 10% polyvinylpyrrolidone iodine (Besetine solution; Hyundai Pharm. Co. Ltd.; Seoul, South Korea) and irrigation with a balanced salt solution (BSS ®; Alcon). Corneal edema in rabbits was clinically evaluated and the animals were sacrificed at 1 day and at 1 and 2 weeks after corneal endothelial injury (n = 3 for each group). The CCT was then measured with a caliper after sacrifice.

### Vital staining

Vital staining was performed using alizarin red S to identify cell borders, and trypan blue to reveal cells with damaged plasma membranes [14]. The endothelial surface was stained with 0.2% trypan blue in 0.9% NaCl for 90 s and the endothelium was stained with 0.2% alizarin red S in 0.9% NaCl (pH 4.2) for 90 s. Excess dye was removed by rinsing with 0.9% NaCl, and the corneas were then fixed for 10 min in 2.5% glutaraldehyde. Following this, the corneas were removed from their supports and trimmed of scleral and conjunctival tissue. The buttons were placed on cavity slides and mounted under a coverslip (which was taped to the slide to flatten the cornea) with a drop of 0.9% NaCl. The endothelium was viewed under transmitted light on a CKX41 microscope (Olympus; Tokyo, Japan) and photographs of the central and peripheral zone of the endothelium were taken. Cells were counted at ×400 magnification.

### Hematoxylin and eosin staining and immunofluorescent staining for Ki67

Enucleated eyes were immediately harvested, corneas were excised from the globe by cutting around the limbus and fresh tissues were fixed in 10% formalin solution followed by embedding in paraffin or were placed into embedding medium (Tissue-Tek OCT Compound; Sakura Finetek USA, Inc.; Torrance, CA, USA).

The tissue sections were cut at a thickness of 3-μm and transferred to glass slides, hydrated and then stained with hematoxylin and eosin. Briefly, after sections were deparaffinized in xylene, and hydrated through a graded series of ethanol, the sections were stained with hematoxylin for 10 min. Following a second wash with running water, the sections were differentiated with 1% HCl in 70% alcohol. Subsequently, the sections were stained with eosin for 1–4 min after washing with running water. Following dehydration and differentiation in alcohol, the sections were mounted on cover slips and observed under light microscopy.

Sections were cut on a cryostat at a thickness of 6-μm and transferred to glass slides using a tape system to avoid potential compression artifacts and allowed to dry at room temperature for 15 min. Immunofluorescence staining of Ki-67 were performed. Slides were fixed for 20 min in 3.7% formaldehyde solution in PBS. The slides were permeabilized for 10 min with 0.5% Triton X-100 and blocked for 1 h with 1% bovine serum albumin at room temperature. After washing, the slides were incubated overnight with a rabbit anti-Ki-67 antibody (Abcam; Cambridge, MA, USA) at 4°C overnight and, then washed with PBS. The cells were incubated with fluorescein isothiocyanate-conjugated rabbit anti-goat antibody or goat anti-rabbit IgG antibody (1:100) for 1 h at 37°C in the dark, then counterstained with Hoechst 33342 dye (1:2000; Molecular Probes; Eugene, OR, USA) to identify the nuclei, in accordance with the manufacturer’s recommendations. Following washing with PBS, the slides were mounted with mounting medium to reduce photobleaching. Negative control staining was conducted in parallel with the omission of primary antibodies.

### Statistical analysis

Data analysis was performed using SPSS software (version 10.0, SPSS, Inc.). Pearson correlation coefficients were calculated to assess the relationship between CT changes and changes in the posterior cornea. The paired *t*-test was used to compare preoperative and postoperative values. The independent sample *t*-test was used to compare parameters between two groups. The statistical significance of experimental differences was evaluated using the paired Student’s t-test, the Student’s t-test, and analysis of variance (ANOVA); p < 0.05 was considered statistically significant.

## Results

### Patients with chemical injury

The clinical study comprised three referral patients (Table 1): a 64-year-old man with chemical injury of the right eye from sulfuric acid (case 1), a 34-year-old man with acute chemical injury of the left eye by the strong alkali, ethylenediamine (case 2), and a 71-year-old woman with chemical injury of the left eye from a strong alkali gum sensitizer (case 3). They showed more than 1/2 limbal ischemia and opaque cornea, which was considered as acute grade IV by Hughes-Roper-Hall classification [15,16]. All three patients underwent paracentesis and irrigation of the anterior chamber immediately upon the first visit. Limbal autograft from the contralateral eye was performed in all injured eyes depending on the severity of the limbal stem cell deficiency 2–4 months after injury. Clinical evaluations occurred immediately after injury and 1, 3, 6, and 9 months later.

**Table 1:** Clinical cases of unilateral chemical eye injury.

	Case 1	Case 2	Case 3
**Age (years)**	64	34	71
**Gender**	Male	Male	Female
**Materials causing chemical injuries of eyes**	Sulfuric acid	Ethylenediamine (strong alkali)	Gum sensitizer (strong alkali)
**Severity (Pfister and Hughes classification)**	Grade IV	Grade IV	Grade IV
**Paracentesis and irrigation of anterior chamber**	Performed	Performed	Performed
**Limbal stem cell deficiency**	Partial (1/2 of total limbus)	Partial (1/2 of total limbus)	Total
**Limbal autograft from the contralateral eye**	Performed	Performed	Performed
**Last visual acuity**	20/30	20/40	20/40

### Corneal edema and opacity in patients with chemical injury

**Fig 1:**
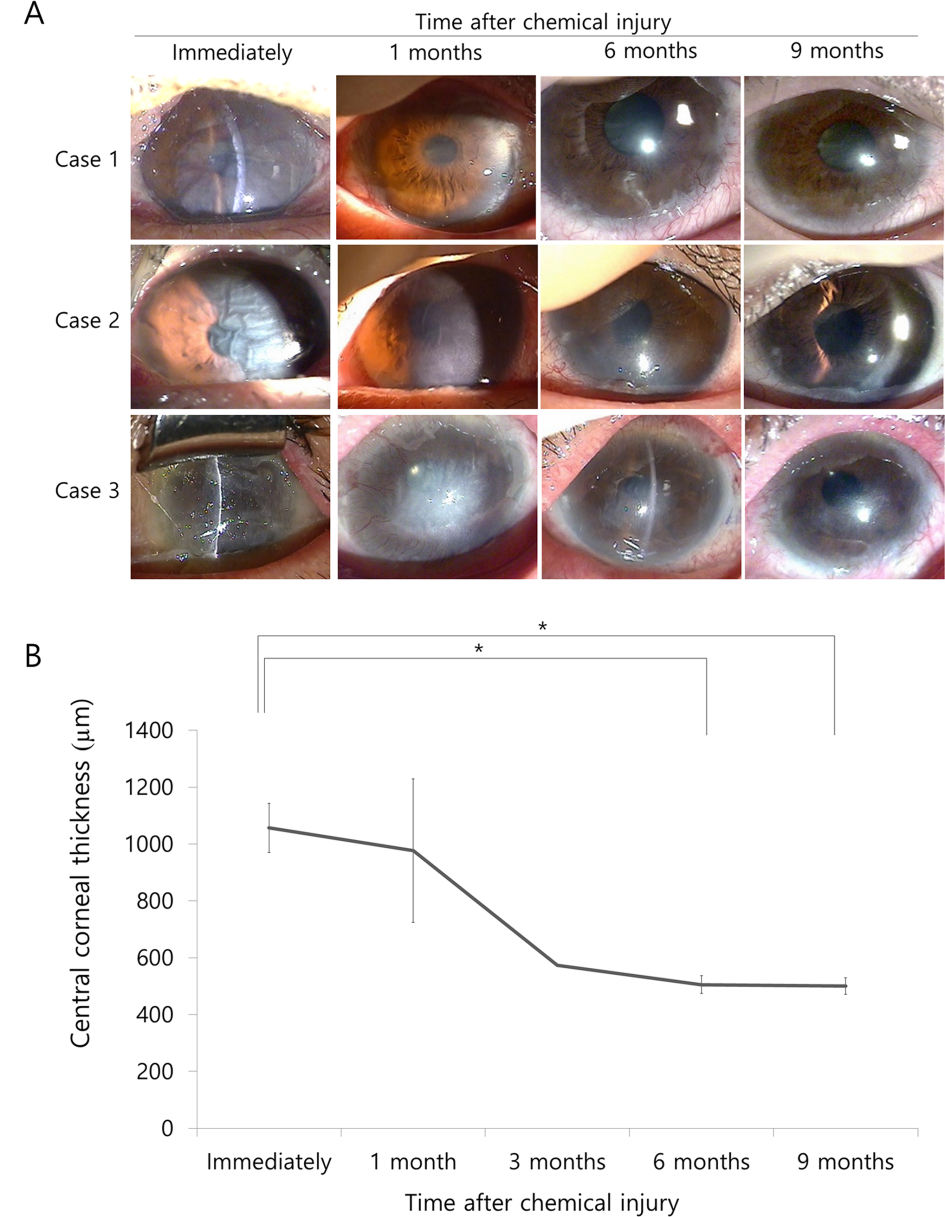
Photographs of anterior segments (A) and central corneal thickness data (CCT; B). A. Chemical injury of the cornea causes severe corneal edema and endothelial damage immediately after chemical injury. Slit lamp examination shows severe edematous and opaque cornea, and Descemet’s membrane folds. Endothelial cells are absent under specular microscopy. Corneal edema is reduced from the periphery to the center over time. Case 1 shows severe corneal edema and Descemet’s membrane fold immediately after injury, central edema and peripheral transparency 1 month after injury, and a clear cornea 6 months after injury. Case 2 shows severe corneal edema and Descemet’s membrane folds immediately after injury; the cornea becomes more transparent from periphery 1 month after injury. Case 3 shows severe corneal edema and Descemet’s membrane folds immediately after injury and the cornea becoming clear from the periphery 1 month after injury. This case also shows transparency of the central edema and periphery 6 months after injury. B. CCT is greater immediately after injury but decreases significantly 6 and 9 months later (p = 0.014 and 0.012, respectively, paired t-test) * Statistically significant by paired t-test

Chemical injury of the cornea caused severe corneal edema and endothelial damage immediately after chemical injury (Fig 1A). Slit lamp examination showed severe edematous, opaque corneas, Descemet’s membrane folds, and more than 1/2 limbal ischemia while specular microscopy revealed an absence of endothelial cells. Corneal edema reduced from the periphery to the center over time. Case 1 showed severe corneal edema, Descemet’s membrane folds and more than 1/2 limbal ischemia immediately after injury, and central edema and peripheral transparency 1 month later. Partial limbal deficiency (1/2 of limbus) occurred and limbo-conjuntival autograft from normal contralateral eye was performed; the cornea was clear 6 months after injury. Case 2 showed severe corneal edema, Descemet’s membrane folds and more than 1/2 limbal ischemia immediately after injury, and the cornea became clear from periphery 1 month later. Partial limbal deficiency (1/2 of limbus) occurred and limbo-conjuntival autograft from normal contralateral eye was performed. Then, the cornea became more transparent and visual acuity improved to 20/30. Case 3 also showed severe corneal edema, Descemet’s membrane folds and total limbal ischemia immediately after injury. The cornea became transparent from the periphery 1 month after injury, and total limbal deficiency was showed. Limbo-conjuntival autograft from normal contralateral eye was performed, and central edema and peripheral transparency occurred 6 months after injury. Visual acuity improved from hand movements to 20/40. CCT measurements by ultrasound pachymetry (Fig 1B, Table 2) were large immediately after injury (1056.7 ± 86.2 μm). CCT was significantly reduced at 6 and 9 months after injury compared to immediately after injury (p = 0.014 and p = 0.012, respectively, paired t-test; Table 2). Raw data may be found in S1 Dataset.

**Table 2:** Specular microscopy study on corneal endothelial cells after chemical injury.

	Eyes with chemical injury (n = 3)	Normal contralateral eyes (n = 3)
**CCT (μm)**		
** Immediately after injury**	1056.7 ± 86.2	535.0 ± 40.6
** One month**	976.5 ± 252.4	544.7 ± 47.4
** Three months**	574.0 ± 0.0	538.7 ± 30.7
** Six months**	505.0 ± 31.7	538.3 ± 32.9
** Nine months**	500.3 ± 29.5	533.0 ± 19.2
**CECD (cells/mm** ^**2**^ **)**		
** Immediately after injury**	0 ± 0	2494.3 ± 672.9
** One month**	0 ± 0	2371.0 ± 632.9
** Three months**	233.0 ± 403.6	2183.3 ± 453.8
** Six months**	814.0 ± 129.7	2174.0 ± 613.3
** Nine months**	1114.0 ± 139.8	2257.7 ± 645.8
**Average cell area (μm** ^**2**^ **)**		
** Immediately after injury**	N/A	423.3 ± 126.9
** One month**	N/A	446.7 ± 138.7
** Three months**	1431 ± N/A	473.7 ± 111.2
** Six months**	1249.7 ± 196.0	490.3 ± 166.0
** Nine months**	907.3 ± 112.0	473.7 ± 162.2

CCT = central corneal thickness; CECD = central endothelial cell density; CV = coefficient of variation; N/A = not available

### Corneal endothelial cell evaluation

**Fig 2:**
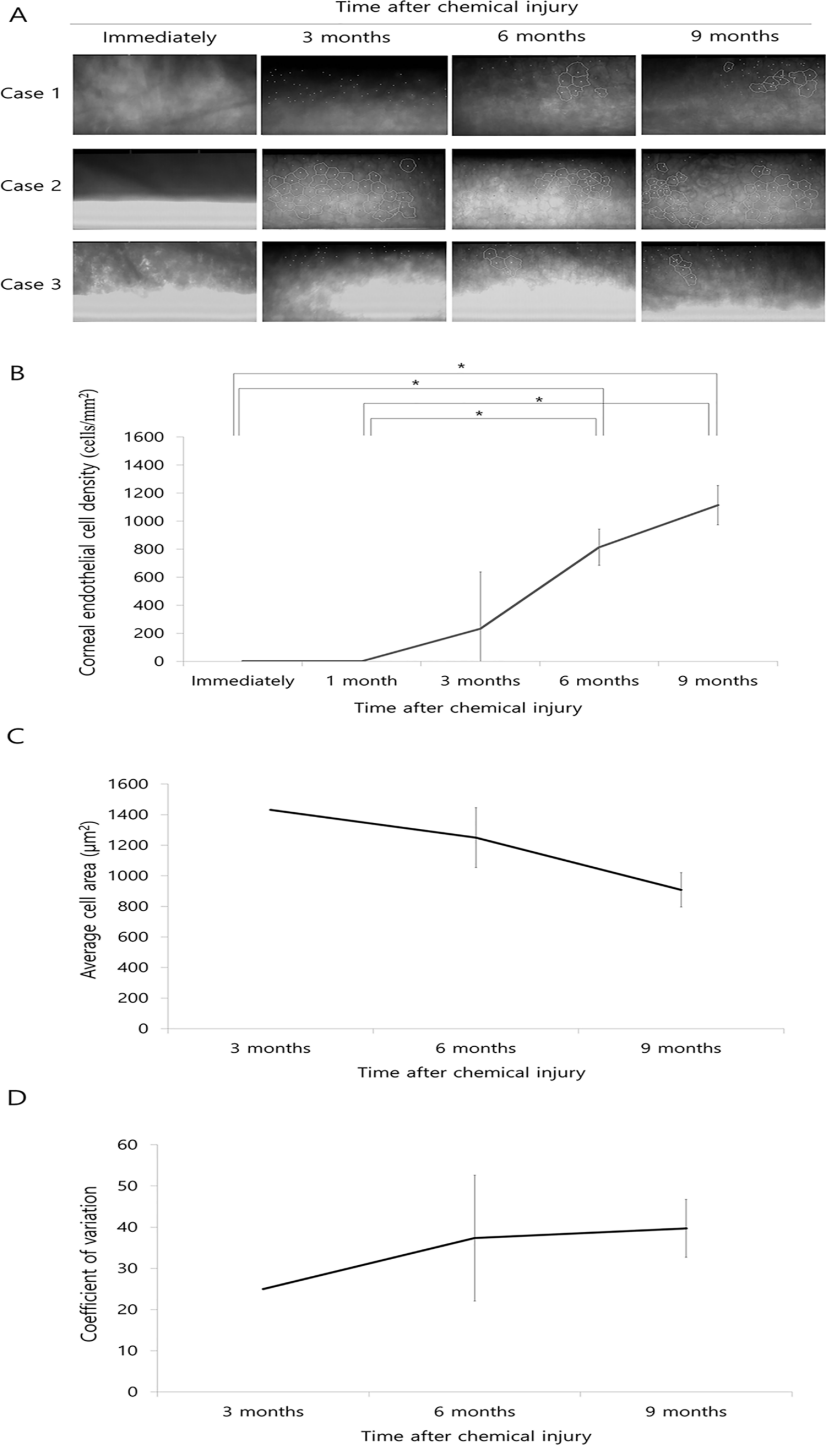
Evaluation of corneal endothelial cells using specular microscopy. A. Corneal endothelial cells are absent immediately after chemical injury; however, they are present 3 months (case 2) or 6 months (case 1 and case 3) later. B. CECD increased significantly 6 months following injury compared to immediately and 1 month after injury (p = 0.008 and p = 0.008, respectively, paired t-test), and 9 months after injury compared to those immediately or 1 months after injury (p = 0.005 and p = 0.005, respectively, paired t-test). C. Average cell area generally decreases over time, although not significantly. D. CV does not change and hexagonal cells are absent 3 to 9 months after injury. * Statistically significant by paired t-test

Corneal endothelial cells were not detected by specular microscopy immediately after chemical injury but were observed at 3 (case 2) or 6 months (case 1 and case 3) post injury. (Fig 2A, Table 2). CECD increased significantly at 6 months post injury compared to immediately and 1 month after injury (p = 0.008 and p = 0.008, respectively, paired t-test; Fig 2B, Table 2) and at 9 months after injury compared to immediately and 1 month after injury (p = 0.005 and p = 0.005, respectively). Average cell area generally decreased over time, although not significantly (Fig 2C). The coefficient of variation (CV) did not change (Fig 2D) and there was no detection of hexagonal cells 3 to 9 months after injury.

### Corneal edema and opacity measurements in the rabbit study

**Fig 3:**
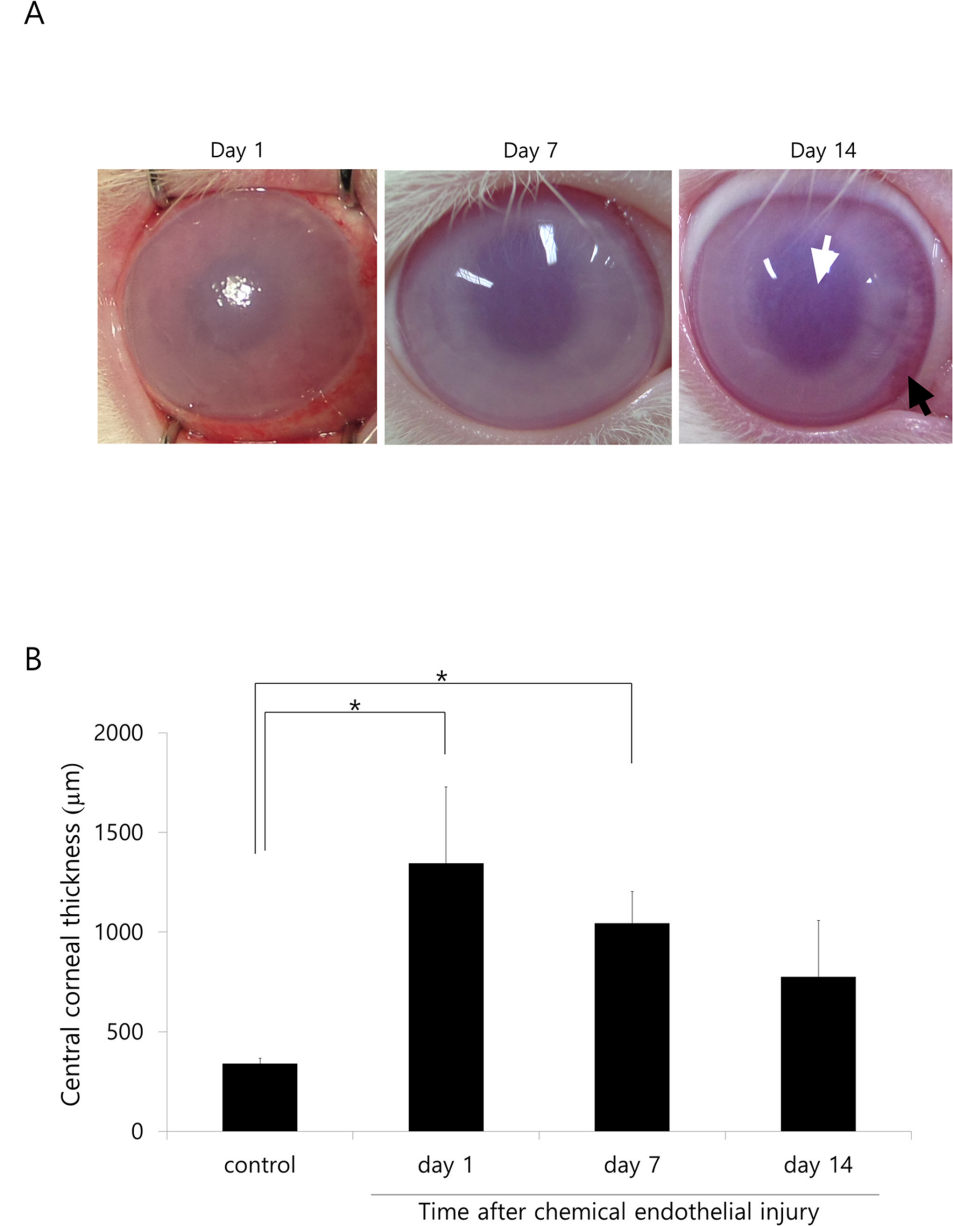
Changes in corneal edema and opacity after chemical corneal endothelial injury in rabbits. A. Corneal edema increases 1 day after injury compared to the control, and then decreases over time; however, corneal opacity does not revert back to levels observed in control rabbits at day 14. Corneal edema decreases from the periphery, and peripheral and central corneas are edematous at day 1 and at day 7. At day 14, the central cornea is edematous (white arrow), while the periphery is transparent (black arrow). B. CCT increases significantly at day 1 and at day 7 after injury compared to the control (p = 0.045 and p = 0.015). CCT generally decreases over time and there are no differences in CCT at day 14 compared to the control, although CCT does not revert to levels observed in control rabbits. * Statistically significant by Student’s t-test

Corneal edema increased significantly 1 day after injury compared to the control and then subsequently decreased over time (Fig 3A); however, corneal opacity did not revert back to levels observed in the control rabbits at day 14. There was a reduction in corneal edema from the periphery; peripheral corneas, as well as those in the center, were edematous at day 1 and day 7. At day 14, corneas in the center were edematous, while those in the periphery were transparent.

CCT increased significantly 1 day after injury compared to the control (p = 0.045; Fig 3B). It then generally decreased over time; there was no difference detected in CCT at day 14 compared to the control, although it did not revert back to levels observed in the control rabbits. Raw data may be found in S2 Dataset.

### Vital staining with alizarin S red and trypan blue in the rabbit study

**Fig 4:**
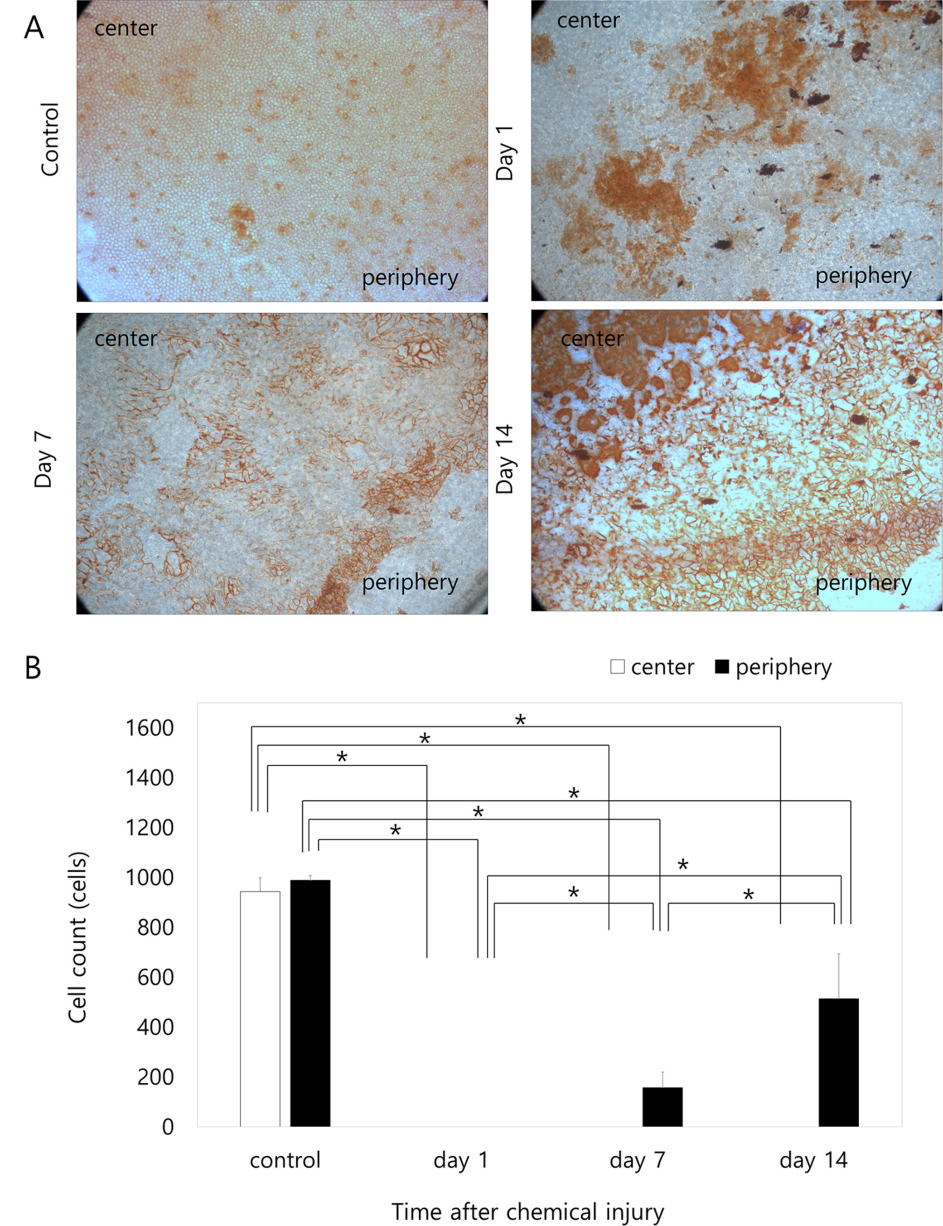
Vital staining of rabbit corneal endothelium with alizarin S red and trypan blue after injection of 0.1 mL of 0.05 N NaOH. A. Corneal endothelial cells (CECs) in both the central and peripheral areas show damage at day 1. At day 7, CECs appeared at the periphery and scanty cells existed at the midperiphery and center 7 days after injury. The peripheral cells began to migrate from the far periphery and to recover from damage. At day 14, the dense small cells were observed at the periphery, the loose large irregular cells appeared at the midperiphery and no cells were still observed at the center of the cornea. The cell integrity of the peripheral cells recovers while cells migrate from the periphery to the center and also recover. B. Cell counts at ×400 magnification. Corneal endothelial cell numbers in the center and in the periphery are significantly lower compared to the control during the entire observation period (p < 0.001 in the center at all time points; p < 0.001 at day 1, p < 0.001 at day 7 and p = 0.012 at day 14 in the periphery). No corneal endothelial cells are present in the periphery or in the center 1 day after injury. Corneal endothelial cells in the periphery increase significantly over time (p < 0.001, one-way ANOVA). Corneal endothelial cells are detected in the periphery 7 days after injury and increase until day 14 (p = 0.022 compared to day 7), although they are lower compared to the number on day 1 (p = 0.014 and p = 0.010). Corneal endothelial cell counts in the center are not observed until day 14. * Statistically significant by Student’s t-test

The CECs in both the central and peripheral area were either damaged, destroyed, or absent at day 1 following injury. Central areas remained in a damaged state without live cells while peripheral areas contained large cells, which had migrated from the far-periphery, and showed signs of recovery 7 days after injury. Fourteen days after injury, the cell integrity of the peripheral cells had been restored and cell migration from the periphery to center for recovery was observed. Peripheral cells had recovered from damage 14 days following injury and, whilst still damaged, central cells showed signs of recovery (Fig 4A). In the periphery, there was a high density of small and polygonal CECs while in the central area, CECs were larger and more irregular in shape. Cell counts at ×400 magnification are presented in Fig 4B. Corneal endothelial cell numbers in the center and in the periphery were significantly lower compared to those in the control during the entire observation period (p < 0.001 in the center at all time points; p < 0.001 at day 1, p < 0.001 at day 7 and p = 0.012 at day 14 in the periphery). Corneal endothelial cells were absent in both the periphery and in the center 1 day after injury. However, corneal endothelial cell numbers in the periphery increased significantly over time (p < 0.001, ANOVA). Corneal endothelial cells were detected in the periphery 7 days after injury and cell numbers were significantly greater at day 14 (p = 0.022) compared to those at day 7; however, cell numbers at day 1 and day 7 were significantly lower compared to day 1 (p = 0.014 and p = 0.010, respectively). Corneal endothelial cells in the central areas were not detected until day 14. Raw data may be found in S3 Dataset.

### Hematoxylin and eosin staining and immunofluorescent staining for Ki67 after chemical injury

**Fig 5:**
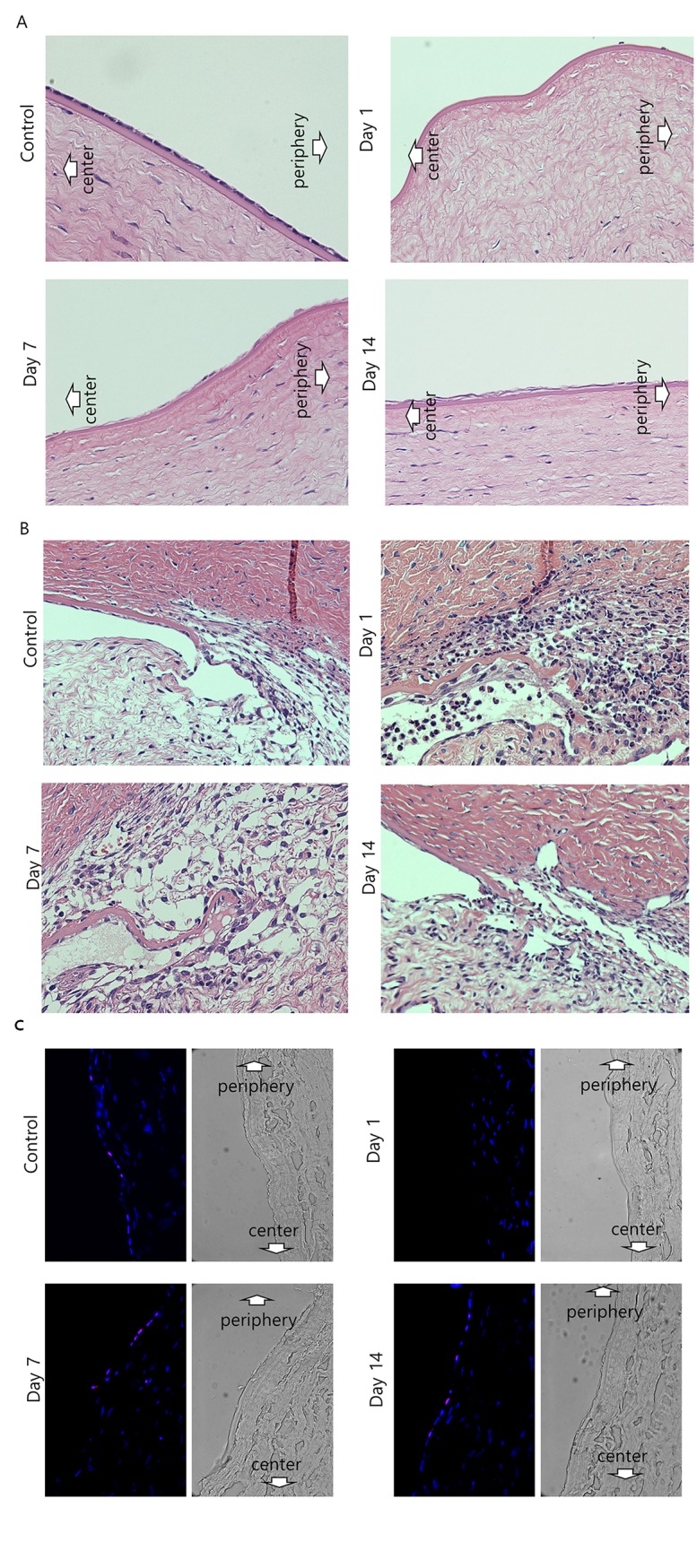
Microscopic images of hematoxylin and eosin staining in corneal endothelium (A) and trabecular meshwork (B), and immunofluorescent staining for the proliferation marker Ki-67 (C). A. With hematoxylin and eosin staining, no cells were observed on the center and periphery of Descemet's membrane 1 day after injury. At day 7, corneal endothelial cells (CECs) were detected at the periphery, but not at the center. At day 14, CECs were detected at the mid-periphery and the periphery. B. With hematoxylin and eosin staining, a lot of inflammatory cells were infiltrated around trabecular meshwork 1 day after injury. At day 7, inflammatory cells disappeared and at day 14, trabecular meshworks were recovered much. C. Ki-67 staining was performed to assess the proliferative cells. Corneal sections were immunostained for Ki-67 (Red) and counterstained with 4',6-diamidino-2-phenylindole (DAPI; blue). Phase contrast images reveals the actual sections. No cells were observed on the center and periphery of Descemet's membrane 1 day after injury. At 7 and 14 days after injury, the cells expressing Ki-67 were observed at the periphery of the cornea. Magnification X 400.

Microscopic images of hematoxylin and eosin stained tissue sections were shown in Fig 5A and 5B. No cells were observed on the center and the periphery of Descemet's membrane 1 day after injury. At day 7, CECs were detected at the periphery, but not at the center. At day 14, CECs were detected at the mid-periphery and the periphery. With hematoxylin and eosin staining, a lot of inflammatory cells were infiltrated around trabecular meshwork 1 day after injury. At day 7, inflammatory cells disappeared and at day 14, trabecular meshworks were recovered much. Ki-67 staining was performed to assess the proliferative cells (Fig 5C). Corneal sections were immunostained for Ki-67 (Green) and counterstained with 4',6-diamidino-2-phenylindole (DAPI; blue). No cells were observed on the center and periphery of Descemet's membrane 1 day after injury. The cells expressing Ki-67 were observed at the periphery of the cornea at 7 and 14 days after injury.

## Discussion

Corneal endothelial cells are essential for corneal transparency [1]. Compensatory cellular hypertrophy, including cell enlargement and migration, has been shown to be essential for corneal endothelial wound healing [1,6]. It has been suggested that there are differences in the CECs between central and peripheral areas of the cornea [17]. However, it is not known how corneal endothelial regeneration occurs after extensive injury. In this study, the healing process and regeneration of corneal endothelium during extensive corneal endothelial injury was investigated in humans and rabbits.

In human cases, severe corneal edema and endothelial damage was observed immediately after severe chemical injury of the cornea and there was an absence of endothelial cells under specular microscopy. However, over time, there was a reduction in corneal edema from the periphery to the center of the cornea, a reduction in CCT, and an increase in CECD. Similarly, corneal edema in rabbits with chemical injury of corneal endothelium increased 1 day after injury followed by a reduction in corneal edema from the periphery over time. Vital staining with alizarin S red and trypan blue did not reveal any cells at the center and at the periphery of the rabbit’s cornea at day 1; however, CECs migrated from the periphery at day 7. At day 14, cells in the periphery were small and regular in shape while cells in the center were large and irregular. This suggests that the CECs in the rabbits proliferated at the periphery and migrated to the center, and implies that cells at the far periphery may survive NaOH exposure. Therefore, cells in the periphery of the cornea could be protected from the chemical injury and may act as a cell reservoir against endothelial damage; however, CECs in rabbits have been shown to be to proliferate differently from humans in vivo [18]. Ki-67 staining was performed to assess the proliferative cells [19]. The proliferation marker Ki-67 were expressed at the periphery 7 and 14 days after injury. This observation is consistent with the suggestion that progenitor cells for CECs may reside at the periphery, between the corneal endothelium and the trabecular meshwork [20,21].

In contrast to corneal epithelial and stromal cells, corneal endothelial wounds usually respond as a compensatory cell hypertrophy in tissue repair rather than cell proliferation [6]. However, the findings from this study suggest that the cells at the posterior limbus can be protected from injury and could be a main source for corneal endothelial wound healing. There are several potential mechanisms underlying this process; first, the cornea is the thickest at the periphery [1], therefore it may be difficult for chemicals to penetrate into the posterior limbus despite being sufficient to cause total limbal deficiency. However, CECs at the periphery survived in spite of 0.05N NaOH injection into anterior chamber in the rabbit study. Secondly, CECs at the posterior limbus may be protected by the iris which is located between the posterior limbus and ciliary body [22]. It has been reported that a small amount of plasma derived protein present in aqueous humor diffuses from the ciliary body stroma to the root of the iris, accumulates in the iris stroma and is then released into the aqueous humor of the anterior chamber [22]. Some of the protein delivered to the iris root immediately enters the trabecular outflow pathways [22]. Thirdly, CECs near trabecular meshwork may be protected because chemicals can wash out into this region [23]. Lastly, it has been suggested that corneal endothelial precursors exist at the post limbus [11,17]. Wounding of the corneas has been reported to activate the production of two additional stem cell markers (Oct-3/4, Wnt-1) as well as two differentiation markers (Pax-6, Sox-2), the latter of which also appears in the corneal endothelial periphery [18]. Stem cells may also reside in the posterior limbus and respond to corneal wounding to initiate an endothelial repair process [11].

The corneal endothelium of rabbits has been known to regenerate and those of human have a limited regenerative capacity in vivo [24]. Human CECs in vivo have been reported to be inhibited in the G1-phase of the cell cycle due to contact inhibition and TGF-beta1 [25]. Several studies have suggested that stem cells exist at the transition zone between trabecular meshwork and corneal endothelium [26].

Previously reported studies for corneal endothelial injury have employed ex vivo model in growth medium using 2–mm scrape wound [27] or in vivo model using cryoinjury on the central cornea [28]. However, in those models, the partial central area of cornea have been damaged. In our model, the extensive corneal endothelial damage using chemical injury was produced. This study revealed firstly that the CECs at the periphery are protected from the extensive endothelial damages by chemical injury in both human and rabbit model. In addition, this study is the first report to reveal the way how the corneal endothelial cells were recovered from periphery to center after extensive corneal endothelial damage.

Furthermore, it has been reported that alkaline agents, in general, penetrate more deeply than acids. The hydroxyl ion causes saponification of fatty acids in cell membranes which results in cellular disruption [29]. Once the epithelium is compromised, alkaline solutions penetrate into the underlying tissues, destroying proteoglycan ground substance and the collagen matrix. Strong alkaline agents penetrate into the anterior chamber and cause widespread inflammation of iris, lens, trabecular meshwork and ciliary body [29,30]. Alkali injuries that reach the trabecular meshwork can damage to the collagen fibrils of the trabecular meshwork, distort trabecular meshwork. Trabecular meshwork distortion and inflammatory debris can lead to increase intraocular pressure [31]. In this study, a lot of inflammatory cells were infiltrated around trabecular meshwork 1 day after injury. At day 7, inflammatory cells disappeared and at day 14, trabecular meshworks were recovered much.

In conclusion, CECs migrated from the periphery to the center of the cornea after corneal endothelial injury. Recovery occurred from CECs in the human posterior limbus; CECs at the periphery may be more protected from injury compared to those in the center. Therefore, the corneal endothelium in the periphery may act as a cell resource for the regeneration of entire corneal endothelium.

## Supporting Information

S1 Dataset Dataset of corneal endothelial cells using specular microscopy. (DOCX)

S2 Dataset Data of central corneal thickness after chemical corneal endothelial injury in rabbits. (DOCX)

S3 Dataset Data of cell count at the center and at the periphery of the cornea after chemical corneal endothelial injury in rabbits. (DOCX)

## References

[1] Joyce NC. Proliferative capacity of the corneal endothelium. Prog Retin Eye Res. 2003;22:359–89. 12852491 10.1016/s1350-9462(02)00065-4

[2] Iwamoto T, Smelser GK. Electron microscopy of the human corneal endothelium with reference to transport mechanisms. Invest Ophthalmol. 1965;4:270–84. 14326614

[3] Edelhauser HF. The balance between corneal transparency and edema: the Proctor Lecture. Invest Ophthalmol Vis Sci. 2006;47:1754–67. 16638979 10.1167/iovs.05-1139

[4] Srinivas SP. Dynamic regulation of barrier integrity of the corneal endothelium. Optom Vis Sci. 2010;87:E239–54. 20142793 10.1097/OPX.0b013e3181d39464 PMC2868144

[5] Engelmann K, Bednarz J, B¨ohnke M. Endothelial cell transplantation and growth behavior of the human corneal endothelium. Ophthalmologe. 1999;96:555–62. 10501982 10.1007/s003470050452

[6] Tamori Y, Deng WM. Compensatory cellular hypertrophy: the other strategy for tissue homeostasis. Trends Cell Biol. 2014;24:230–7. 24239163 10.1016/j.tcb.2013.10.005 PMC4022146

[7] Honda H, Ogita Y, Higuchi S, Kani K. Cell movements in a living mammalian tissue: long-term observation of individual cells in wounded corneal endothelia of cats. J Morphol. 1982;174:25–39. 7143447 10.1002/jmor.1051740104

[8] Ikebe H, Takamatsu T, Itoi M, Fujita S. Changes in nuclear DNA content and cell size of injured human corneal endothelium. Exp Eye Res. 1988;47:205–15. 3409992 10.1016/0014-4835(88)90004-8

[9] Bredow L, Schwartzkopff J, Reinhard T. Host-Derived Endothelial Regeneration of Corneal Transplants in a Rat Keratoplasty Model. Ophthalmic Res. 2014;52:60–4. 24993185 10.1159/000360739

[10] Bredow L, Schwartzkopff J, Reinhard T. Regeneration of corneal endothelial cells following keratoplasty in rats with bullous keratopathy. Mol Vis. 2014;20:683–90. 24883013 PMC4037534

[11] Kanai A, Tanaka M, Ishii R, Nakajima A. Bullous keratopathy after anterior-posterior radial keratotomy for myopia for myopic astigmatism. Am J Ophthalmol. 1982;93:600–6. 7081358 10.1016/s0002-9394(14)77375-1

[12] Wagoner MD. Chemical injuries of the eye: current concepts in pathophysiology and therapy. Surv Ophthalmol. 1997;41:275–313. 9104767 10.1016/s0039-6257(96)00007-0

[13] Mimura T, Yamagami S, Amano S. Corneal endothelial regeneration and tissue engineering. Prog Retin Eye Res. 2013;35:1–17. 23353595 10.1016/j.preteyeres.2013.01.003

[14] Taylor MJ, Hunt CJ. Dual staining of corneal endothelium with trypan blue and alizarin red S: importance of pH for the dye-lake reaction. Br J Ophthalmol. 1981;65:815–9. 6172144 10.1136/bjo.65.12.815 PMC1039687

[15] Hughes W. Alkali burns of the eye; review of the literature and summary of present knowledge. Arch ophthalmology. 1946;35: 423–49. 10.1001/archopht.1946.00890200430010 21027052

[16] Roper-Hall MJ. Thermal and chemical burns. Trans Ophthalmol Soc U K. 1965; 85: 631–53. 5227208

[17] He Z, Campolmi N, Gain P, Ha Thi BM, Dumollard JM, Duband S, et al Revisited microanatomy of the corneal endothelial periphery: new evidence for continuous centripetal migration of endothelial cells in humans. Stem Cells. 2012;30:2523–34. 22949402 10.1002/stem.1212

[18] Van Horn DL, Sendele DD, Seideman S, Buco PJ. Regenerative capacity of the corneal endothelium in rabbit and cat. Invest Ophthalmol Vis Sci. 1977;16:597–613. 873721

[19] Guinebretière JM, Sabourin JC. Ki-67, marker of proliferation. Ann Pathol. 1997;17:25–30. 9162153

[20] Hara S, Hayashi R, Soma T, Kageyama T, Duncan T, Tsujikawa M, et al Identification and potential application of human corneal endothelial progenitor cells. Stem Cells Dev. 2014;23:2190–201. 24588720 10.1089/scd.2013.0387

[21] McGowan SL, Edelhauser HF, Pfister RR, Whikehart DR. Stem cell markers in the human posterior limbus and corneal endothelium of unwounded and wounded corneas. Mol Vis. 2007;13:1984–2000. 17982423

[22] Freddo TF. A contemporary concept of the blood-aqueous barrier. Prog Retin Eye Res. 2013;32:181–95. 23128417 10.1016/j.preteyeres.2012.10.004 PMC3544162

[23] Swaminathan SS, Oh DJ, Kang MH, Rhee DJ. Aqueous outflow: Segmental and distal flow. J Cataract Refract Surg. 2014;40:1263–72. 25088623 10.1016/j.jcrs.2014.06.020 PMC4151118

[24] Van Horn DL, Sendele DD, Seideman S, Buco PJ. Regenerative capacity of the corneal endothelium in rabbit and cat. Invest Ophthalmol Vis Sci. 1977; 16:597–613. 873721

[25] Joyce NC, Harris DL, Mello DM. Mechanisms of mitotic inhibition in corneal endothelium: contact inhibition and TGF-beta2. Invest Ophthalmol Vis Sci. 2002;43:2152–9. 12091410

[26] He Z, Campolmi N, Gain P. Revisited microanatomy of the corneal endothelial periphery: new evidence for continuous centripetal migration of endothelial cells in humans. Stem Cells. 2012;30:2523–34. 22949402 10.1002/stem.1212

[27] Senoo T, Joyce NC. Cell cycle kinetics in corneal endothelium from old and young donors. Invest Ophthalmol Vis Sci. 2000;41:660–7. 10711678

[28] Okumura N, Koizumi N, Kay EP, Ueno M, Sakamoto Y, Nakamura S, et al The ROCK inhibitor eye drop accelerates corneal endothelium wound healing. Invest Ophthalmol Vis Sci. 2013;54:2493–502. 23462749 10.1167/iovs.12-11320

[29] Wagoner MD. Chemical injuries of the eye: current concepts in pathophysiology and therapy. Surv Ophthalmol. 1997;41:275–313. 9104767 10.1016/s0039-6257(96)00007-0

[30] Lin A, Patel N, Yoo D, Demartelaere S, Bouchard C. Management of ocular conditions in the burn unit: thermal and chemical burns and stevens-johnson syndrome/toxic epidermal necrolysis. J Burn Care Res. 2011;32:547–60. 21799437 10.1097/BCR.0b013e31822b0f29

[31] Eslani M, Baradaran-Rafii A, Movahedan A, Djalilian AR. The ocular surface chemical burns. J Ophthalmol. 2014;2014:196827. 25105018 10.1155/2014/196827 PMC4106115

