# Supplementary material for: Recovery of Corneal Endothelial Cells from Periphery after Injury
Source: PLoS One. 2015 Sep 17;10(9):e0138076. doi: 10.1371/journal.pone.0138076 (PMC4574742; doi:10.1371/journal.pone.0138076)
Supplement: S1 Dataset — (DOCX) [file pone.0138076.s001.docx]

**Dataset S1. Dataset of corneal endothelial cells using specular microscopy.**

|  | **Case 1** | | **Case 2** | | **Case 3** | |
| --- | --- | --- | --- | --- | --- | --- |
|  | Eyes with chemical injury | Normal contralateral eyes | Eyes with chemical injury | Normal contralateral eyes | Eyes with chemical injury | Normal contralateral eyes |
| CCT (μm) |  |  |  |  |  |  |
| Immediately after injury | 1002 | 580 | 1012 | 524 | 1156 | 501 |
| One month | N/A | 522 | 798 | 523 | 1155 | 599 |
| Three months | N/A | 519 | 575 | 523 | 574 | 574 |
| Six months | 502 | 515 | 539 | 524 | 476 | 576 |
| Nine months | 500 | 525 | 530 | 524 | 471 | 520 |
| CECD (cells/mm^2^) |  |  |  |  |  |  |
| Immediately after injury | 0 | 3039 | 0 | 2624 | 0 | 1766 |
| One month | 0 | 2835 | 0 | 2628 | 0 | 1650 |
| Three months | 0 | 2468 | 699 | 2422 | 0 | 1660 |
| Six months | 693 | 2513 | 951 | 2543 | 798 | 1466 |
| Nine months | 989 | 2637 | 1088 | 2624 | 1265 | 1512 |
| Average cell area (μm^2^) |  |  |  |  |  |  |
| Immediately after injury | N/A | 323 | N/A | 381 | N/A | 566 |
| One month | N/A | 353 | N/A | 381 | N/A | 606 |
| Three months | N/A | 406 | 1431 | 413 | N/A | 602 |
| Six months | 1444 | 396 | 1052 | 393 | 1253 | 682 |
| Nine months | 1013 | 379 | 919 | 381 | 790 | 661 |

CCT = central corneal thickness; CECD = central endothelial cell density; CV = coefficient of variation; N/A = not available
