# Supplementary material for: Recovery of Corneal Endothelial Cells from Periphery after Injury
Source: PLoS One. 2015 Sep 17;10(9):e0138076. doi: 10.1371/journal.pone.0138076 (PMC4574742; doi:10.1371/journal.pone.0138076)
Supplement: S2 Dataset — (DOCX) [file pone.0138076.s002.docx]

**Dataset S2. Data of central corneal thickness after chemical corneal endothelial injury in rabbits**.

|  | **Central corneal thickness (µm)** | | |
| --- | --- | --- | --- |
|  | Rabbit A | Rabbit B | Rabbit C |
| Control | 420 | 380 | 370 |
| Day 1 | 1830 | 1250 | 1130 |
| Day 7 | 910 | 1160 | 1210 |
| Day 14 | 1100 | 770 | 570 |
