# Supplementary material for: Recovery of Corneal Endothelial Cells from Periphery after Injury
Source: PLoS One. 2015 Sep 17;10(9):e0138076. doi: 10.1371/journal.pone.0138076 (PMC4574742; doi:10.1371/journal.pone.0138076)
Supplement: S3 Dataset — (DOCX) [file pone.0138076.s003.docx]

**Dataset S3. Data of cell count at the center and at the periphery of the cornea after chemical corneal endothelial injury in rabbits**.

|  | Cell count at the center | Cell count at the periphery |
| --- | --- | --- |
| Control |  |  |
| 1 | 960 | 960 |
| 2 | 992 | 992 |
| 3 | 864 | 1008 |
| 4 | 960 | 992 |
| Day 1 |  |  |
| 1 | 0 | 0 |
| 2 | 0 | 0 |
| 3 | 0 | 0 |
| 4 | 0 | 0 |
| Day 7 |  |  |
| 1 | 0 | 208 |
| 2 | 0 | 68 |
| 3 | 0 | 172 |
| 4 | 0 | 184 |
| Day 14 |  |  |
| 1 | 0 | 744 |
| 2 | 0 | 320 |
| 3 | 0 | 450 |
| 4 | 0 | 542 |
